# Difference in Serum Levels of Vitamin D Between Canalolithiasis and Cupulolithiasis of the Horizontal Semicircular Canal in Benign Paroxysmal Positional Vertigo

**DOI:** 10.3389/fneur.2019.00176

**Published:** 2019-03-01

**Authors:** Takafumi Nakada, Saiko Sugiura, Yasue Uchida, Hirokazu Suzuki, Masaaki Teranishi, Michihiko Sone

**Affiliations:** ^1^Department of Otorhinolaryngology, National Center for Geriatrics and Gerontology, Obu, Japan; ^2^Department of Otorhinolaryngology, Aichi Medical University, Nagakute, Japan; ^3^Department of Otorhinolaryngology, Nagoya University, Nagoya, Japan

**Keywords:** benign paroxysmal positional vertigo, vitamin D, canalolithiasis, geotropic, cupulolithiasis, apogeotropic

## Abstract

**Background and Purpose:** In the horizontal canal benign paroxysmal positional vertigo (BPPV), cupulolithiasis shows apogeotropic direction changing nystagmus lasting more than 1 min, while canalolithiasis leads to geotropic direction changing nystagmus lasting < 1 min. The difference between cupulolithiasis and canalolithiasis is widely accepted to be the attachment of the displaced otoconia to the cupula of a semicircular canal. Several studies have shown a relationship between BPPV and vitamin D deficiency, but no studies have compared serum levels of vitamin D between canalolithiasis and cupulolithiasis patients. The purpose of this study was to clarify the difference in vitamin D serum level between canalolithiasis and cupulolithiasis of the horizontal canal.

**Methods:** This retrospective study included 20 and 15 patients with canalolithiasis and cupulolithiasis of the horizontal canal, respectively. Serum levels of 25-hydroxyvitamin D [25(OH)D] during the acute phase of BPPV were measured.

**Results:** The mean 25(OH)D serum level in patients with canalolithiasis and cupulolithiasis was 13.2 ± 1.4 and 20.4 ± 1.6 ng/mL, respectively, and the difference was statistically significant (*p* = 0.0014), also after adjusting for age and sex (*p* = 0.0351). Eighteen out of 20 (90%) and 5 of 15 (33%) patients were diagnosed with vitamin D deficiency in the canalolithiasis and cupulolithiasis groups, respectively, and this difference was also statistically significant (*p* = 0.0005).

**Conclusion:** We found that serum vitamin D level in patients with canalolithiasis was significantly lower than that in patients with cupulolithiasis of the horizontal canal.

## Introduction

Benign paroxysmal positional vertigo (BPPV) is the most frequent vestibular disorder (1). It is caused by the abnormal stimulation of the cupula, upon changing head position, by otoconia, either floating or attached to the cupula, in any of the three semicircular canals. The Dix-Hallpike maneuver and the supine roll test are used to diagnose BPPV, and patients are treated through specific canalith-repositioning maneuvers, although remission can be expected within several days also without treatment (2). Among the semicircular canals, the posterior canal is the most affected for anatomical reasons. In the horizontal semicircular canal, canalolithiasis and cupulolithiasis exhibit the characteristic nystagmus: canalolithiasis leads to geotropic direction changing nystagmus lasting <1 min, while cupulolithiasis shows apogeotropic direction changing nystagmus lasting more than 1 min, in the supine roll test (3). In addition, when the otoconia fall into the short arm and directly onto the cupula, apogeotropic horizontal nystagmus are observed (short-arm canalolithiasis) (4). No nystagmus may be provoked in the contralateral spine position because the otoconia may fall out during the spine roll test in the contralateral direction. Moreover, rapid transition from apogeotropic to geotropic direction changing nystagmus during the supine roll test may be observed when otoconia are located in the anterior part of the horizontal canal (3).

BPPV patients are at a high risk of fracture, associated with abnormal bone turnover (5). Vitamin D mainly controls the absorption of calcium and phosphate from the small intestine, which plays a crucial role in bone turnover (6). On the other hand, vitamin D deficiency can change the structure of the otoconia, which are made of calcium carbonate (7). Such structural changes may induce otoconia to easily detach from the otolith organ, leading to BPPV attacks. No studies have compared serum levels of vitamin D between canalolithiasis and cupulolithiasis patients, which we focused on.

The purpose of this study was to clarify the difference in vitamin D serum level between canalolithiasis and cupulolithiasis of the horizontal canal. The results will contribute to explain the pathophysiological difference between canalolithiasis and cupulolithiasis.

## Materials and Methods

### Participants

Patients who visited the National Center for Geriatrics and Gerontology between May 2017 and September 2018 and were diagnosed with horizontal canal canalolithiasis or cupulolithiasis, according to the criteria established by the Barany Society (3) were included in the study. Blood samples were collected when the patients visited the hospital outpatient department with a vertigo attack. The serum level of 25-hydroxyvitamin D [25(OH)D], the main indicator of vitamin D status, was measured from the samples using a chemiluminescence immunoassay (BML Inc., Japan).

### Statistical Analyses

Statistical analysis was carried out with the Statistical Analysis System (SAS) package, version 9.3 (SAS Institute, Cary, NC, USA). The difference in the mean level of serum 25 hydroxyvitamin D between the groups was assessed using a general linear model to adjust for age and sex. A serum 25(OH)D level of <20 ng/mL was defined as vitamin D deficiency. The relationship between the diagnosis (canalolithiasis or cupulolithiasis) and vitamin D deficiency (<20 ng/mL) was assessed using the chi squared test and logistic regression. The level of significance was set at *p* < 0.05.

## Results

A total of 38 patients were diagnosed with BPPV of the horizontal semicircular canal. Of these, 35 patients, followed up until the symptoms and nystagmus disappeared, were enrolled in the study, while 3 were excluded due to loss of follow up. Among the 35 participants, 20 were diagnosed with horizontal canal canalolithiasis (CAN), and 15 with horizontal canal cupulolithiasis (CUP). The age of the CAN group was 76.7 ± 7.1 years, with 17 females (85%), while that of the CUP group was 75.9 ± 5.4 years, with 6 females (40%). The difference in sex prevalence was statistically significant (*p* = 0.0055, Table 1A). The mean serum 25(OH)D level of in the CAN group was 13.2 ± 1.4 ng/mL [least square (LS) mean ± standard error (SE)] and that in the CUP group was 20.4 ± 1.6 ng/mL (LS means ± SE), a statistically significant difference (*p* = 0.0014, Table 2). After adjusting for age only, sex only, and for both using a general linear model, the level of 25(OH)D in the CAN group was still significantly lower than in the CUP group (*p* = 0.0018, *p* = 0.0274, and *p* = 0.0351, respectively, Table 2).

**Table 1A:** Number, sex, and age of the participants.

	**Canalolithiasis**	**Cupulolithiasis**	***p*-value**
Number	20	15	
Female/male	17/3	6/9	0.0055^*^
Age, mean ± SD	76.7 ± 7.1	75.9 ± 5.4	0.695$5_{*}^{*}$

SD, standard deviation.

* Chi squared test;

${\;}_{*}^{*}$t-test.

**Table 1B:** Number of canalolithiasis/cupulolithiasis patients diagnosed with vitamin D deficiency. Vitamin D deficiency (serum level < 20 ng/mL) was associated with the onset of a canalolithiasis (chi squared test, *p* = 0.0005).

	**Canalolithiasis**	**Cupulolithiasis**
Vitamin D deficiency	18	5
No vitamin D deficiency	2	10

**Table 2:** Serum level of vitamin D in patients with canalolithiasis and cupulolithiasis, and the covariates used in the general linear model.

**Moderator variable**	**Serum level of vitamin D in *canalolithiasis* (ng/mL), LS means ± SE**	**Serum level of vitamin D in *cupulolithiasis* (ng/mL), LS means ± SE**	***p*-value**
no (base model)	13.2 ± 1.4	20.4 ± 1.6	0.0014
age	13.2 ± 1.4	20.4 ± 1.6	0.0018
sex	14.7 ± 1.6	19.9 ± 1.5	0.0274
both age and sex	14.8 ± 1.6	19.9 ± 1.5	0.0351

LS, least square means; SE, standard error.

*Serum vitamin D levels were significantly lower in patients with canalolithiasis than those with cupulolithiasis using general linear model even adjusting by age, sex, or both*.

Vitamin D deficiency was diagnosed in 18 out of 20 patients (90%) in the CAN group and 5 out of 15 (33%) in the CUP group, and such difference was statistically significant (*p* = 0.0005; Table 1B).

## Discussion

Several studies have reported the association of BPPV with vitamin D deficiency and impaired bone turnover (8–12), without however differentiating between canalolithiasis and cupulolithiasis. We showed that vitamin D serum level is lower in canalolithiasis of the horizontal canal than in cupulolithiasis of horizontal canal. Moreover, the number of female patients and of patients with vitamin D deficiency were higher in the CAN group compared to the CUP group. Females are more likely than males to be affected by vitamin D deficiency (13), but the significance of the difference between the two pathologies was preserved after statistically adjusting for sex.

Our previous study demonstrates that the patients with BPPV have a higher risk of fracture, calculated using the Fracture Risk Assessment Tool (FRAX) (5). Calcium is the main nutrient needed for bone development, and vitamin D controls the small bowel absorption of calcium and potassium. Serum levels of vitamin D are directly related to bone mineral turnover (6).

Otoconia are made from an inorganic calcium carbonate, calcite, deposited onto an organic matrix (14), therefore vitamin D deficiency can affect the structure of otoconia. Kao et al. extracted particulate matter from the posterior semicircular canal of two patients during posterior canal occlusion surgery, and examined them with scanning electron microscopy. They demonstrated that both intact and degenerate otoconia, most of which are interconnected with linking fibrils, associated with particulate matter within the semicircular canal of BPPV patients (15). Further study is needed to evaluate the effects of vitamin D deficiency on the structure of otoconia.

One limitation of this study is the relatively small sample size, which might compromise the generality of the results. However, the statistical significance of the results obtained with such a modest sample size demonstrates that the effect we revealed is quite sizeable.

In conclusion, the present study showed significantly lower vitamin D serum levels in patients with canalolithiasis of horizontal canal compared to those with cupulolithiasis, and vitamin D deficiency in 90% of the canalolithiasis patients. These results suggest the pathophysiological difference between canalolithiasis and cupulolithiasis. In patients with BPPV, and especially with canalolithiasis, a possible vitamin D deficiency should be considered. The early detection of osteoporosis or osteopenia in patients with BPPV would prevent osteoporotic fractures and BPPV recurrence. Further research is however needed to assess the generality of our results.

## Ethics Statement

This retrospective study was approved by the Ethical Standards Committee of the National Center for Geriatrics and Gerontology (No. 1187). Informed consent was obtained by the opt-out method on the center's website (16, 17).

## Author Contributions

TN designed the study, collected, and analyzed the data, and wrote the manuscript. SS and YU analyzed and interpreted part of the data. HS collected part of the data. MT and MS revised the manuscript.

### Conflict of Interest Statement

The authors declare that the research was conducted in the absence of any commercial or financial relationships that could be construed as a potential conflict of interest.
